# Are common names becoming less common? The rise in uniqueness and individualism in Japan

**DOI:** 10.3389/fpsyg.2015.01490

**Published:** 2015-10-21

**Authors:** Yuji Ogihara, Hiroyo Fujita, Hitoshi Tominaga, Sho Ishigaki, Takuya Kashimoto, Ayano Takahashi, Kyoko Toyohara, Yukiko Uchida

**Affiliations:** ^1^Department of Cognitive Psychology in Education, Graduate School of Education, Kyoto UniversityKyoto, Japan; ^2^Japan Society for the Promotion of ScienceTokyo, Japan; ^3^Department of Human Coexistence, Graduate School of Human and Environmental Studies, Kyoto UniversityKyoto, Japan; ^4^Faculty of Education, Kyoto UniversityKyoto, Japan; ^5^Kokoro Research Center, Kyoto UniversityKyoto, Japan

**Keywords:** uniqueness, name, individualism, cultural change, cultural product, individualization, Japan

## Abstract

We examined whether Japanese culture has become more individualistic by investigating how the practice of naming babies has changed over time. Cultural psychology has revealed substantial cultural variation in human psychology and behavior, emphasizing the mutual construction of socio-cultural environment and mind. However, much of the past research did not account for the fact that culture is changing. Indeed, archival data on behavior (e.g., divorce rates) suggest a rise in individualism in the U.S. and Japan. In addition to archival data, cultural products (which express an individual’s psyche and behavior outside the head; e.g., advertising) can also reveal cultural change. However, little research has investigated the changes in individualism in East Asia using cultural products. To reveal the dynamic aspects of culture, it is important to present temporal data across cultures. In this study, we examined baby names as a cultural product. If Japanese culture has become more individualistic, parents would be expected to give their children unique names. Using two databases, we calculated the rate of popular baby names between 2004 and 2013. Both databases released the rankings of popular names and their rates within the sample. As Japanese names are generally comprised of both written Chinese characters and their pronunciations, we analyzed these two separately. We found that the rate of popular Chinese characters increased, whereas the rate of popular pronunciations decreased. However, only the rate of popular pronunciations was associated with a previously validated collectivism index. Moreover, we examined the pronunciation variation of common combinations of Chinese characters and the written form variation of common pronunciations. We found that the variation of written forms decreased, whereas the variation of pronunciations increased over time. Taken together, these results showed that parents are giving their children unique names by pairing common Chinese characters with uncommon pronunciations, which indicates an increase in individualism in Japan.

## Introduction

Cultural psychology has revealed substantial cultural variation in human psychology and behavior, emphasizing the mutual construction of socio-cultural environment and mind (Kitayama and Cohen, 2007; Heine, 2011). However, most previous research compared psychology and behavior only at a single point in time, which did not account for the fact that culture is dynamic and changing (see Greenfield, 2009). Considering that cultural psychology is a discipline that aims to explore how culture and mind make each other up (Shweder, 1990), cultural change should also be examined. Investigating how cultures change and how people adapt to such changes can contribute to cultural psychology, which leads to a better understanding of human nature.

### Two Windows to Investigate Cultural Change

Cultural change has been demonstrated empirically with analyses of archival data. For example, Gentile et al. (2010) did a meta-analysis of scores on the Rosenberg Self-Esteem Scale (Rosenberg, 1965) between 1988 and 2008 among American middle school, high school, and college students. They showed that self-esteem had increased among adolescents and young adults in the U.S., caused by the cultural emphasis on self-worth over time.

However, examining archival data has limitations because it is not always the case that same items have been used consistently over the years. Hence, research has started to focus on cultural products as a desirable means of investigating cultural change. Cultural products are defined as “tangible, public representations of culture such as advertising or popular texts” (Morling and Lamoreaux, 2008, p. 199). Psyches and behaviors are reflected in cultural products, so cultural products enable researchers to investigate psyches and behaviors outside the head. Moreover, cultural products are tangible aspects of culture, so they persist over time. For instance, Twenge et al. (2012, 2013) examined how frequently individualistic words/phrases and first singular pronouns, which are thought to reflect individualistic tendency (Kashima and Kashima, 1998), have been used in books written in American English from 1960 to 2008, and found that American culture has become more individualistic. Similarly, the frequency of individualistic words in books also indicated the urbanization and cultural change toward greater individualism in the U.S. and UK (Greenfield, 2013). Researchers have used not only books but also other cultural products, such as song lyrics (DeWall et al., 2011) and dictionary (Oishi et al., 2013), to examine cultural change.

### Cultural Change toward Greater Individualism in Japan

Has Japan experienced a cultural shift toward greater individualism as European–American cultures have? Past research repeatedly showed that cultures in Europe and North America have become more individualistic (e.g., Gentile et al., 2010; Twenge et al., 2012, 2013; Greenfield, 2013). Yet, few studies have examined empirically whether East Asian cultures have become more individualistic.

Similar to other countries, Japan has been affected by globalization. In particular, Japan has a strong political and economic relationship with the U.S., so Japanese culture has been tremendously influenced by American culture. Hence, individualistic values and systems have flowed into Japanese society. For example, Japanese companies have imported a performance-based salary system that is based on individualistic concepts (e.g., Japan Productivity Center, 2013), and schools have put more emphasis on children’s uniqueness and independence (e.g., Doi, 2004). Moreover, it has been suggested that economic wealth promotes individualism (e.g., Inglehart and Baker, 2000; Greenfield, 2009; Grossmann and Varnum, 2015). If individuals have sufficient wealth, the need for interdependence should relatively decrease. Japan experienced gradual economic growth after World War II. Although the rate of economic growth slowed after 1990s, relative wealth has still increased (e.g., inflation-adjusted GDP per capita has increased). Hence, it is expected that Japanese culture has gradually become more individualistic.

Secondary analysis of archival data has shown that Japanese culture became more individualistic, at least in some respects^1^ from the 1950 to 2000s (Hamamura, 2012). For example, family size decreased, divorce rate increased, and the ratio of people who live in urban areas increased.

However, little research has investigated cultural change concerning individualism in East Asia, which makes it unclear whether East Asian culture has become more individualistic. As we noted above, using archival data to describe cultural change has its limitations, but these limitations can be overcome by examining cultural products. Conducting investigations from different approaches with different variables should increase the generalizability of the previous finding (Hamamura, 2012). Moreover, to reveal dynamic aspects of culture, it is important to present temporal data across cultures. Therefore, it is necessary to investigate changes in individualism, not only in European–American cultures but also in East Asian cultures.

### Names as an Index of Individualism

The present research focuses on the names of people as an index of individualism. Names are rich sources of information. They can signal gender, ethnicity, or class (Kasof, 1993). In addition, names affect psychological, social, and economic consequences (for a review, see Christenfeld and Larsen, 2008). For example, people with easy-to-pronounce names are judged positively and are associated with social success (the name-pronunciation effect; Laham et al., 2012). Moreover, parents’ values or orientations are reflected in the first names they choose for their children (Lieberson and Bell, 1992). Thus, names can be regarded as a behavioral measure of important parental choices and decisions, not only for their babies, but also for themselves.

In individualistic cultures, uniqueness is important (e.g., Kim and Markus, 1999). Previous research demonstrated that the uniqueness of children’s first names is a valid index of individualism. Varnum and Kitayama (2011) conducted a state-level analysis of the relationship between the rate of common names and individualistic tendency, and found that common names are given less frequently to children in more individualistic frontier states in both the U.S. and Canada. In other words, unique names are more likely to be given in more individualistic states (e.g., those in the Mountain West and the Pacific Northwest). Furthermore, they indicated that common names are given less frequently (i.e., unique names are given more frequently) in more individualistic countries^2^.

If a culture has become more individualistic, the rate of parents who give common names to their children should decrease. In fact, prior research showed that in the U.S., the rate of giving common names to babies decreased from 1880 to 2007, suggesting an increase in uniqueness and individualism^3^ in the U.S. (Twenge et al., 2010).

### Present Research

In the present research, we investigated whether Japanese culture has become more individualistic by examining changes in names as a cultural product. We predicted that, consistent with previous research (especially the results of behavioral measures; Hamamura, 2012), Japanese culture has become more individualistic. Specifically, we hypothesized that the rate of parents who give their children common names would have decreased over time.

Due to the Japanese writing and reading system, we examined names using three indices and these indices were examined separately. As most Japanese names are written using Chinese characters (*kanji*), the first index is the combination of Chinese characters (e.g., the combination of “大” and “翔” to create “大翔”). In addition, each Chinese character (e.g., “大” and “翔”) has a meaning (e.g., “大” represents big or large, and “翔” represents to fly or a wing), so each Chinese character was also examined for the second index. For the third index, we examined the verbal pronunciation of the name. This is because even if the same Chinese character is used, the verbal pronunciation or reading of that character can differ, as many Chinese characters used in Japan (i.e., *kanji*) have multiple possible pronunciations. For instance, the possible pronunciations for “大翔” include “Hiroto,” “Haruto,” “Yamato,” and “Tsubasa.” This is because parents can freely assign a pronunciation to any given Chinese character (this practice is legally permitted). Each Chinese character has a common or typical pronunciation, but parents can provide an uncommon pronunciation other than the typical pronunciation. Thus, it is necessary to examine not only Chinese characters but also their pronunciations.

We predicted that unique names would have increased over time. Yet, there were three ways of giving unique names in the Japanese cultural context. Hence, we conducted an examination to determine which strategy was more prevalent. In the first way, parents can give both unique written Chinese characters and a unique pronunciation, whereby uniqueness is expressed both in writing and reading. In the second way, parents can give a name with common pronunciation, but which is written with unique Chinese characters. In other words, uniqueness is expressed in writing rather than in reading. In order to make names unique, it is not always necessary to give both unique Chinese characters and unique pronunciations. Even if the pronunciation is same, if it is written with different characters, the name should be unique. This is similar to the different spellings of common names in the European–American cultural context (e.g., Caitlin, Caitlyn, Kaitlin, Kaitlyn, Kaitlynn, Katelyn, and Katelynn). In the third way, parents can give a common written name that has a unique pronunciation. That is, parents give common Chinese characters but read them in a unique way. We should consider each component (i.e., Chinese characters, pronunciation) holistically as a name, especially in cultures that have a writing system based on Chinese characters. Even if the same Chinese character is used, the readings of that character can be different because many Chinese characters used in Japan (i.e., *kanji*) have multiple possible pronunciations. This practice is different from the European–American cultural practice, in that the written characters do not always lead to the same pronunciation of that character. Parents can freely assign a pronunciation to a given Chinese character. Each Chinese character has its standard or common pronunciation, but parents can give an uncommon pronunciation that differs from the usual pronunciation. Therefore, giving a name comprised of common Chinese characters but an uncommon reading is sufficient to make it unique.

Unlike in the U.S. where the government (the Social Security Administration) publishes a database capturing nearly the entire population (Twenge et al., 2010; Social Security Administration, 2013), it was impossible to collect name data for the entire population of babies in Japan. Hence, we used data from private companies that collected the names of newborns each year. In Japan, two such databases were available. One is from the Benesse Corporation, which deals with goods and services for babies and their parents. The other is from the Meiji Yasuda Life Insurance Company, which deals with insurance. The sample size of the Benesse data was large, but that of Meiji Yasuda was relatively small. Thus in Study 1, we investigated whether names have become more unique in Japan by examining the Benesse data. Moreover, in Study 2 we replicated Study 1 using the Meiji Yasuda data, which were collected independently of Study 1.

## Study 1

### Method

#### Data

Data was retrieved from the Benesse Corporation’s website which is publicly available (Benesse Corporation, 2013). They collect baby names from customers who bought their products, such as toys, books, and nursery items. They published rankings of the most common first names (and their rates of use) given to newborns each year over 9 years, from 2005 to 2013^4^. Sample sizes in each year are shown in **Table 1**. Approximately 40,000 names of newborns were collected each year during this period. Currently, about 1,000,000 babies are born in Japan each year (Ministry of Health, Labour and Welfare, 2014a). Therefore, a sample size of 40,000, which is the equivalent of 4% of the entire population of interest, is large.

**Table 1:** Sample sizes in Study 1 and 2.

		2004	2005	2006	2007	2008	2009	2010	2011	2012	2013	Average
Study 1	Boys	**—**	17,170	24,998	18,821	19,564	19,401	19,706	17,959	17,275	15,720	18,957
	Girls	**—**	16,222	24,126	17,723	18,369	17,939	18,752	16,541	16,365	14,652	17,854
	Total	**—**	33,392	49,124	36,544	37,933	37,340	38,458	34,500	33,640	30,372	36,811
Study 2	Boys	4,861	4,292	4,409	4,591	4,621	4,595	4,078	3,648	3,388	5,338	4,382
	Girls	4,419	4,082	4,167	4,204	4,275	4,254	3,805	3,503	3,222	5,026	4,096
	Total	9,280	8,374	8,576	8,795	8,896	8,849	7,883	7,151	6,610	10,364	8,478

#### Name Indices

We calculated the rate of popular given names each year in Japan. As we noted earlier, due to the Japanese writing and reading system, we examined three indices (the combination of Chinese characters, individual Chinese characters, and verbal pronunciation) separately. Concerning the combination of Chinese characters, the company published a ranking of the top 100 most common combinations of Chinese characters^5^. In line with prior works (Twenge et al., 2010; Varnum and Kitayama, 2011), the top 10 ranking was used in the present study. In terms of individual Chinese characters, the company displayed the top 50 most common Chinese characters in 2005, the top 100 from 2006 to 2008, and the top 20 from 2009 to 2012. To examine changes over time and compare results among the three indices, the top 10 names were used in our analysis. With regard to verbal pronunciation, the company published the top 100 most common pronunciations from 2005 to 2008 and the top 20 from 2009 to 2013. To examine changes over time and compare results among three indices, we used changes in the top 10 rankings as an index of change^6^.

#### Strategy of Analysis

We followed procedures used in the earlier work by Hamamura (2012) to distinguish whether common names are increasing, decreasing, or not changing in prevalence. First, we computed an ecological correlation (correlation with year) that indicated a linear trend for each index over time. In this calculation, each score was weighted by the sample size of its respective year to give a proportionally higher weight to the samples with more names than those with fewer names (e.g., Gentile et al., 2010). As a conventional criterion, indices with correlations below |0.10| were operationalized as showing no change^7^ (Hamamura, 2012). Furthermore, we calculated the annual change, which captures the absolute magnitude of change. Even if high ecological correlations are found, if the absolute magnitude of change is small, this should be interpreted as showing a lack of change. In other words, we have to consider not only linear trends, but also the slope of change. We conducted a simple regression analysis predicting each score by year (i.e., *y* = *Bx* + *c*, where *y* = each score of the name index, *B* = the unstandardized regression coefficient, *x* = the year, and *c* = the regression constant). We used unstandardized regression coefficients as rates of annual change^8^. As with ecological correlation, indices with annual rates of less than 0.1% were operationalized as showing no change (Hamamura, 2012). In addition, we inspected a scatter plot of the relation between year and each name index to check whether other patterns of change were present (e.g., U-shaped).

In addition, to check whether temporal changes of common names are an indicator of uniqueness/individualism in Japan, we tested whether changes in unique name rates were related to changes in previously validated indices of individualism. Previous research developed the Japanese Collectivism Scale (JCS; Yamawaki, 2012) which measures prefectural-level (similar to state-level in the U.S.) collectivism by modeling Vandello and Cohen (1999)’s American Collectivism Index. This scale is constructed of five indicators (divorce to marriage ratio, percentage of elderly living alone, percentage of nuclear family households, percentage of people living alone, percentage of three-generation households) and was validated both at the individual and prefectural level. This scale was also used to examine cultural change in Japan (Hitokoto and Tanaka-Matsumi, 2014). Following previous research, we calculated the annual JCS for each year from 2005 to 2013, and computed the correlations between each index and the JCS^9^. The data on divorce to marriage ratio was obtained from the Ministry of Health, Labour and Welfare (2014a), and other data were obtained from the Ministry of Health, Labour and Welfare (2014b). In line with the prior work (Hamamura, 2012), correlations of more than |0.30| was used as a conventional criterion.

### Results

#### Combinations of Chinese Characters

**FIGURE 1:**
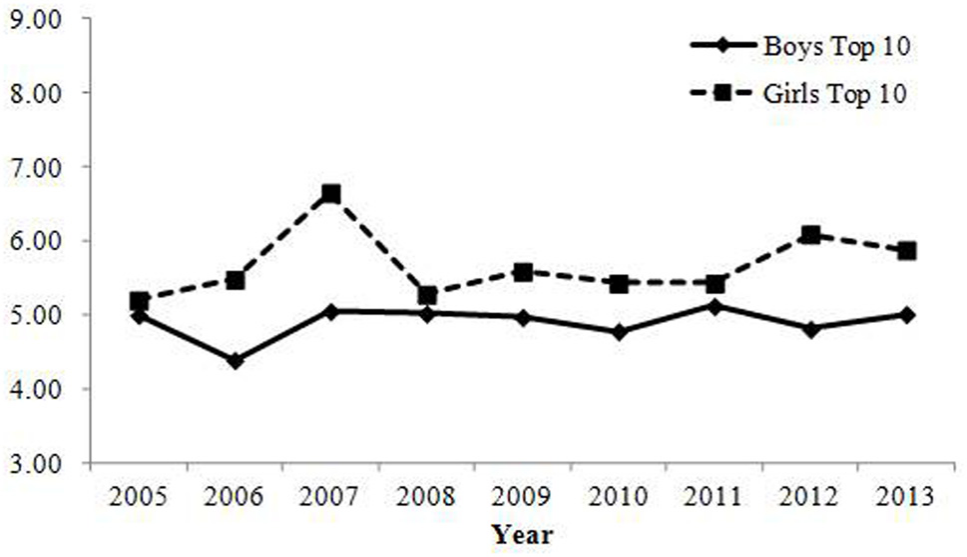
**Percentage of babies receiving names with one of the top 10 most common combinations of Chinese characters in Study 1.** Changes were small in magnitude, showing that the rate of parents who give common combinations of Chinese characters has not changed over time.

Changes in the percentage of parents who gave the top 10 most common combinations of Chinese characters are shown in **Table 2**; **Figure 1**^10^. Correlations with the year were positive, but their changes were small in magnitude. Therefore, the results revealed that the rate of parents who chose common combinations of Chinese characters did not change over time.

**Table 2:** Average rates (%), correlations with year (*r*), and annual changes (%) of top 10 common names in Study 1 and 2.

		Study 1			Study 2		
		Average	Correlation	Annual	Average	Correlation	Annual
		rate (%)	with year (*r*)	change (%)	rate (%)	with year (*r*)	change (%)
Combination of Chinese characters	Boys	4.91	0.31	0.03	4.77	0.22	0.04
	Girls	5.67	0.21	0.04	5.48	0.02	0.00
Individual Chinese characters	Boys	50.01	**0.32**	**0.10**	—	—	—
	Girls	54.62	**0.80**	**0.39**	—	—	—
Pronunciation	Boys	16.23	**-0.83**	**-0.18**	14.77	**-0.59**	**-0.11**
	Girls	13.54	**-0.88**	**-0.16**	13.53	0.29	0.09

*Hyphens mean there were no data available from database. Correlations with year and annual changes were calculated by weighting sample sizes. Numbers in bold letters represent scores that are beyond the criteria both in correlation with year (more than |0.10|) and annual change (more than 0.1%), which indicate changes.*

#### Individual Chinese Characters

**FIGURE 2:**
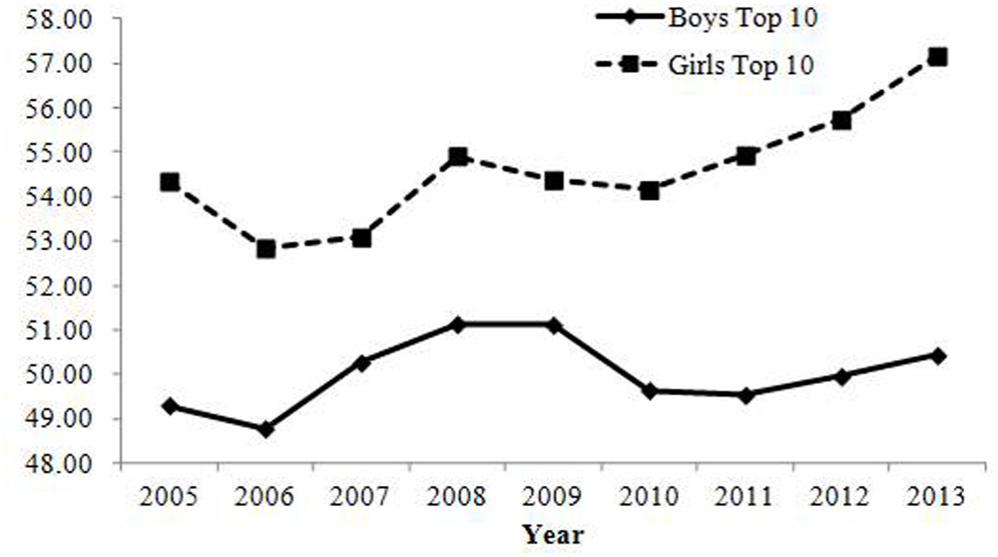
**Percentage of babies receiving names with one of the top 10 most common Chinese characters (individual character) in Study 1.** The rate of babies with common Chinese characters has increased and changes were sufficiently large in magnitude.

Changes in the percentage of parents who gave the top 10 most common Chinese characters are shown in **Table 2**; **Figure 2**. Both correlations were positive and annual changes were sufficiently large^11^. Thus, the results indicated that the rate of parents giving common Chinese characters increased over the years.

#### Pronunciation

**FIGURE 3:**
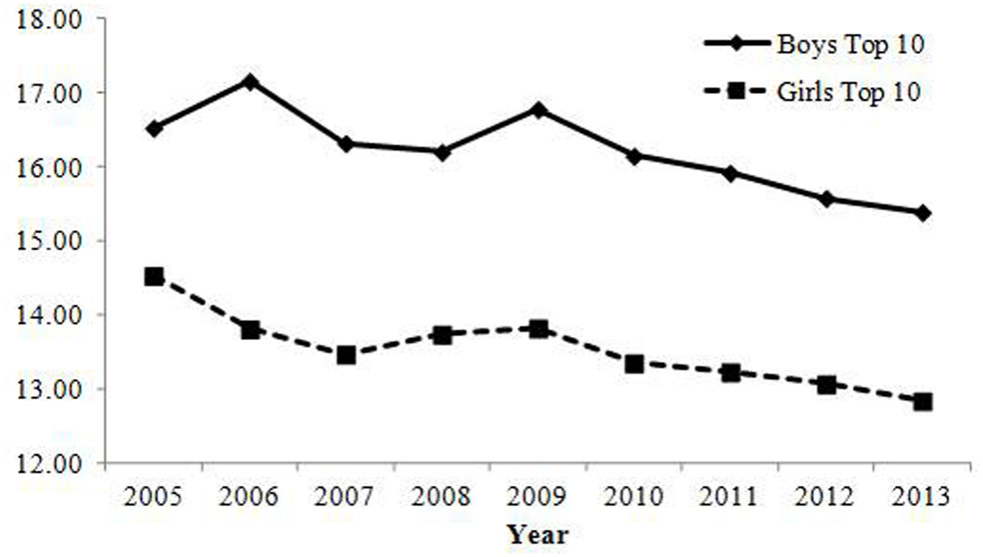
**Percentage of babies receiving names with one of the top 10 most common pronunciations in Study 1.** The rate of babies with common pronunciation has decreased and changes were sufficiently large in magnitude.

Changes in the percentage of parents who gave the top 10 most common pronunciations are shown in **Table 2**; **Figure 3**. Both correlations were negative and annual changes were sufficiently large, showing that the rate of parents who gave common pronunciations decreased over the years.

#### Controlling for Confounding Factors

The rate of common names might be associated with other factors other than individualism. For instance, it has been shown that economic wealth promotes individualism (e.g., Inglehart and Baker, 2000; Greenfield, 2009; Grossmann and Varnum, 2015). In fact, past research indicated that GDP per capita was negatively correlated with the rate of common names (Varnum and Kitayama, 2011). Economic wealth and individualism are related with each other, but they are conceptually different. Furthermore, parents might spend more time choosing names for their children when they have only one child than they do when they have two or three children.

First, we computed simple correlations between the year, GDP per capita (inflation adjusted; Cabinet Office, Government of Japan, 2014), birth rate (Ministry of Health, Labour and Welfare, 2014a) and each of the three naming indicators (**Table 3**). We found that GDP per capita was negatively related to the rates of the common pronunciations, regardless of gender. This result was consistent with the idea that economic wealth promotes individualism (e.g., Inglehart and Baker, 2000; Greenfield, 2009; Grossmann and Varnum, 2015) and the past finding (Varnum and Kitayama, 2011). In addition, birth rate was positively associated with the rate of common pronunciations.

**Table 3:** Simple and partial correlations between year, GDP per capita, birth rate, and each name index in Study 1.

	Combination of Chinese characters		Individual Chinese characters		Pronunciation	
			
	Boys	Girls	Boys	Girls	Boys	Girls
Year	0.31	0.21	**0.32**	**0.80**	**-0.83**	**-0.88**
GDP per capita	-0.06	0.60	-0.30	0.27	**-0.53**	**-0.64**
Birth rate	-0.40	-0.16	-0.01	-0.78	**0.77**	**0.51**
Year (GDP per capita and birth rate were controlled)	0.06	0.07	**0.54**	**0.54**	**-0.60**	**-0.98**
GDP per capita (Year and birth rate were controlled)	-0.26	0.59	-0.44	-0.14	**-0.40**	**-0.93**
Birth rate (Year and GDP per capita were controlled)	-0.32	0.11	0.31	-0.48	0.38	-0.87

*Each score was calculated by weighting sample size. Numbers in bold letters represent scores that are beyond the criterion (more than |0.30|) for both genders in a consistent direction. Year was negatively related to the rate of the top 10 most common pronunciations regardless of gender, even when GDP per capita and birth rate were controlled. Year was also positively associated with the rate of the top 10 most common Chinese characters regardless of gender even after two other variables were controlled. GDP per capita was negatively related to the rate of the top 10 most common pronunciations.*

Next, we calculated partial correlations between the year and each of the three naming indices while controlling for GDP per capita and birth rate (**Table 3**). The pattern of the results was consistent with the original results obtained from simple correlations for pronunciation and individual Chinese characters. In terms of common combinations of Chinese character, the absolute magnitude of correlations became smaller and was below the criterion.

#### Relationships with Indices of Individualism

We computed correlations between each index and the JCS (**Table 4**)^12^. Results indicated that the rates of the top 10 most common pronunciations in both boys’ and girls’ names were positively related to the JCS. That is, giving names with popular pronunciations was associated with collectivism.

**Table 4:** Correlation coefficients between each index of name and Japanese Collectivism Scale (JCS; Yamawaki, 2012) in Study 1 and 2.

	Combination of Chinese characters		Individual Chinese characters		Pronunciation	
			
	Boys	Girls	Boys	Girls	Boys	Girls
Study 1	-0.41	-0.20	-0.21	–0.75	**0.79**	**0.80**
Study 2	-0.22	-0.02	—	—	0.47	–0.35

*Hyphens mean there were no data available from database. Correlations were calculated by weighting sample sizes. Numbers in bold letters represent scores that are beyond the criterion (more than |0.30|) for both genders in a consistent direction.*

In contrast, the rates of the top 10 most common combinations of Chinese characters for boys were negatively associated with the JCS. This pattern ran contrary to that which should have been expected (i.e., that the phenomenon of many parents using common combinations of Chinese characters in children’s names should be indicative of collectivism). Thus, giving common combinations of Chinese characters for boys cannot be seen as an index of collectivism^13^. The rates of the top 10 most common combinations of Chinese characters for girls were not associated with the JCS.

The rates of the top 10 most common individual Chinese characters was not associated with the JCS for boys and was negatively associated with the JCS for girls. This pattern for girls was contrary to that which should have been expected (i.e., that the phenomenon of many parents using common Chinese characters in children’s names should be considered as a sign of collectivism). Thus, giving names with common Chinese characters cannot be seen as an index of collectivism^14^.

These results demonstrated that only the rates of the top 10 most common pronunciations can be seen as an index of collectivism.

### Discussion

We found that the rate of parents who gave common pronunciations decreased. Further, this index was positively correlated with the previously validated collectivism index. In contrast, the rate of parents who gave common Chinese characters increased, but this index was not associated with the previously validated collectivism index. These results demonstrated that uniqueness as an aspect of individualism has increased over the years in Japan. Further, these changes remained prominent even when GDP per capita and birth rate were controlled.

## Study 2

### Method

In order to replicate the findings of Study 1, we analyzed changes in baby names from another large-sample survey similarly as in Study 1.

#### Data

Data was retrieved from the Meiji Yasuda Life Insurance Company’s website which is publicly available (Meiji Yasuda Life Insurance Company, 2013). They collected baby names from people holding insurance policies with the company. Thus, the sample was different from that in Study 1^15^ (data from educational service company). Sample sizes for each year are shown in **Table 1**. They collected about 8,000 names of newborns each year.

#### Name Indices

We examined two indices for names: the combination of Chinese characters and the pronunciation (the ranking of each Chinese character was not released in this data set). The top 100 combinations of Chinese characters and the top 50 pronunciations were available for each year. As in Study 1, we used the top 10 rankings.

In addition, Meiji Yasuda offers two kinds of information that were not available from Study 1 samples. First, we assessed the number of writing variations for the top 3 most common pronunciations. For example, a given name pronounced as “Yui” was written in 24 variations (such as “結衣,” “優衣,” and “唯”) in the 2013 samples of girls’ names. We counted how many variations were represented among the top 3 most common pronunciations. Second, we assessed the number of pronunciation variations for the top 10 most common combinations of Chinese characters in each year. For instance, “大翔” was pronounced with six variations (such as “Hiroto,” “Daito,” “Haruto,” “Yamato,” “Taiga,” and “Masato”) in the 2013 samples of boys’ names. We counted how many variations were included among the top 10 most common combinations of Chinese characters in each year. Further, we assessed the number of names that had more than two different pronunciation variants among the top 10 (some combinations of Chinese characters were read in only one fashion; e.g., “蓮 (Ren)” and “颯太 (Souta)” in the 2013 samples of boys’ names).

### Results

#### Combination of Chinese Characters

**FIGURE 4:**
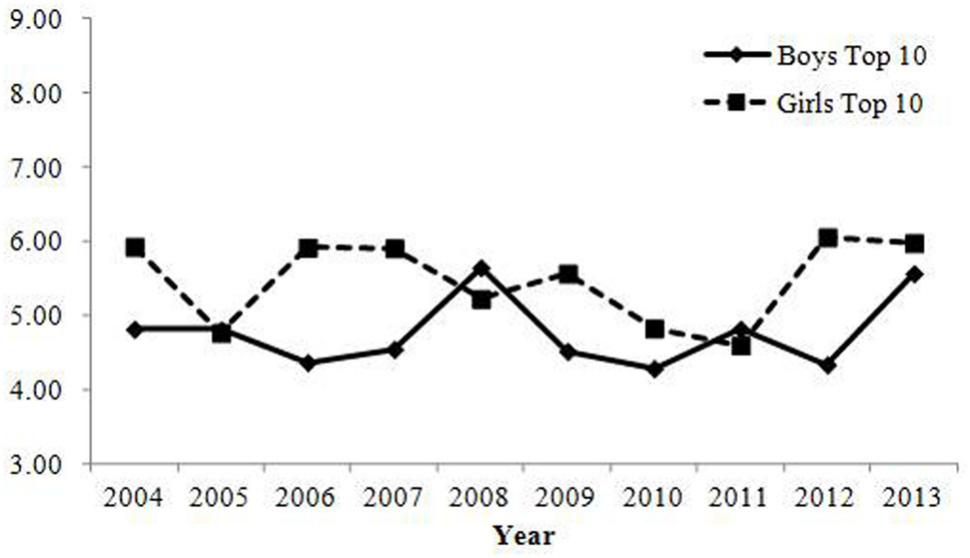
**Percentage of babies receiving names with one of the top 10 most common combinations of Chinese characters in Study 2.** Changes were small in magnitude, showing that the rate of parents who give common combinations of Chinese characters has not changed over time.

Changes in the percentage of parents who gave one of the top 10 most common combinations of Chinese characters are shown in **Table 2**; **Figure 4**. No indices reached the criteria for change with regard to ecological correlation and annual change. Therefore, consistent with Study 1, the results showed that the rate of parents who gave common combinations of Chinese characters did not change over time.

#### Pronunciation

**FIGURE 5:**
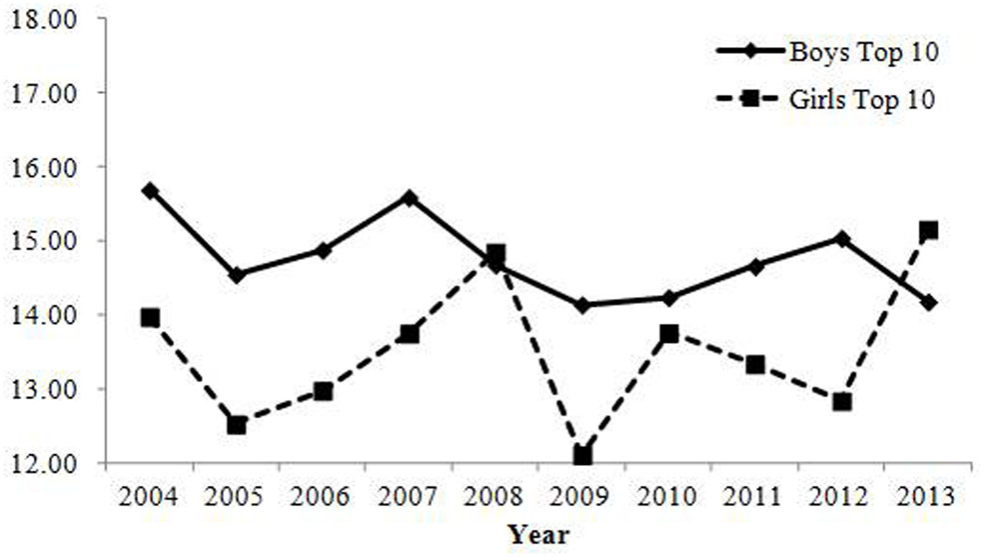
**Percentage of babies receiving names with one of the top 10 most common pronunciations in Study 2.** The rate of babies with common pronunciation in boys has decreased and changes were sufficiently large in magnitude. In contrast, the correlation between year and the rate of babies with common pronunciations in girls were below the criterion. However, it should be noted that there were greater fluctuations due to the inclusion of many duplicated rankings.

Changes in the percentage of parents who gave one of the top 10 most common pronunciations are presented in **Table 2**; **Figure 5**. Negative correlations in boys’ names revealed that the rate of parents who gave common pronunciations decreased, which is consistent with Study 1. In contrast to Study 1, there was not a negative correlation between year and the rate of the top 10 most common pronunciations and declining annual change for girls’ names. As shown in **Figure 5**, there were greater fluctuations for girls’ names relative to boys’ names. Indeed, there were 13 variations of names within the top 10 because of duplicated rankings (two 5th and five 9th ranked names) in 2013, 12 names in 2008 and 2011, and 11 names in 2004, 2007, and 2010. This was not the case for the boys’ names (11 variations in 2004, 2007 and 2012) and in Study 1 (not at all for boys’ names; only one case for girls’ names in 2005 with 11 variations). The rates of the top 10 most common pronunciations for girls were lower than those for boys (this was the case for both Study 1 and 2), and the sample sizes for girls were slightly smaller than those for boys. Hence, the possibility of having duplicated rankings due to the relatively small sample sizes was higher for girls’ names than for boys’ names. These reasons might lead to this inconsistency in the results for girls’ names between Study 1 and 2.

#### Controlling for Confounding Factors

As in Study 1, partial correlations between year and each name index were computed, controlling for GDP per capita (inflation adjusted; Cabinet Office, Government of Japan, 2014) and birth rate (Ministry of Health, Labour and Welfare, 2014a). Concerning the combination of Chinese characters, partial correlations were consistent with the result obtained from simple correlations (*r*_boy_ = 0.21, *r*_girl_ = 0.11). In terms of pronunciation, partial correlation between year and rate of common pronunciations was consistent with the result obtained from a simple correlation for boys’ names (*r* = -0.43). In contrast, the partial correlation was positive for girls’ names (*r* = 0.47). This inconsistency was probably caused by the inclusion of more than 10 variations among the top 10 rankings, as we mentioned above.

#### Relationships with Indices of Individualism

As in Study 1, we calculated the correlations between each index and the JCS (**Table 4**). Results showed that the rates of the top 10 pronunciations in boys’ names were positively related to the JCS, but those in girls’ names were negatively related to the JCS (this inconsistency may also have arisen because of the inclusion of more than 10 pronunciation variations in the top 10 rankings). In contrast, the rates of the top 10 most common combinations of Chinese character were not associated with the JCS, regardless of gender.

#### Variations in the Written Form of Common Pronunciations

**FIGURE 6:**
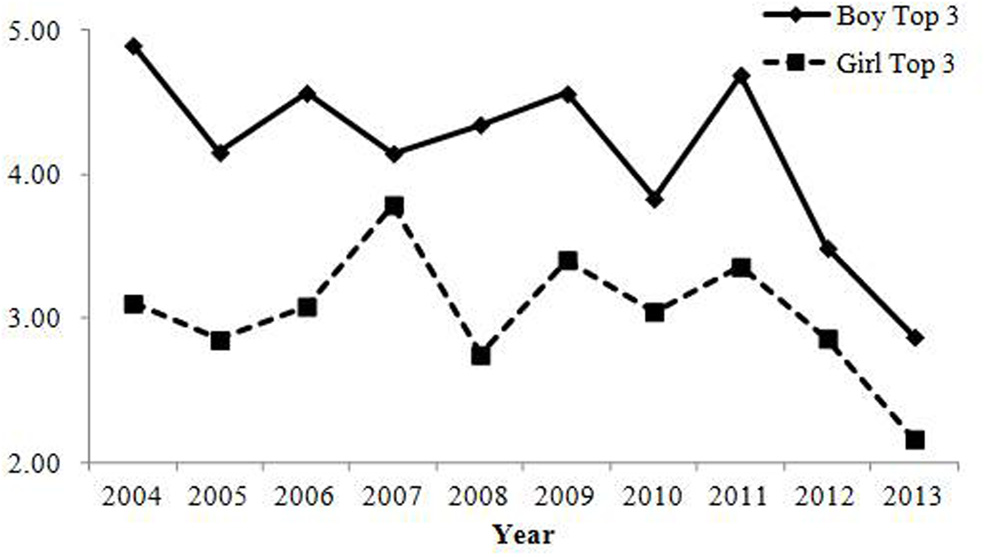
**Square average variations of written forms for names with one of the top 3 most common pronunciations in Study 2.** Variations of written form decreased over time for both in boys’ and girls’ names.

Changes in how much the written form of the top 3 most common pronunciations varied are shown in **Table 5**; **Figure 6**. Both for boys and girls, correlations between the year and the square average variation were negative, suggesting that parents tend not to seek uniqueness in the written form of names.

**Table 5:** Variations of writings in the top 3 most common pronunciations (Study 2).

		2004	2005	2006	2007	2008	2009	2010	2011	2012	2013	Correlation with year (*r*)
Boys	Total variation^1^	140	111	128	117	128	128	100	119	87	95	
	Average variation^2^	0.51	0.47	0.49	0.44	0.45	0.49	0.44	0.56	0.42	0.27	**-0.58**
	Square average variation^3^	4.90	4.16	4.57	4.15	4.35	4.56	3.83	4.69	3.49	2.87	**-0.72**
Girls	Total variation^1^	75	65	73	75	68	83	72	79	60	56	
	Average variation^2^	0.39	0.38	0.39	0.41	0.33	0.42	0.39	0.44	0.41	0.25	**-0.34**
	Square average variation^3^	3.11	2.85	3.09	3.79	2.75	3.41	3.05	3.36	2.86	2.16	**-0.41**

*Correlations were calculated by weighting sample sizes. Numbers in bold letters in the “correlation with year” column represent scores that are beyond the criterion (more than |0.10|). ^1^Total number of writing variations included in the top 3 most common pronunciations. ^2^Total variation divided by the number of babies who have the top 3 most common pronunciations. ^3^Total variation divided by square root of number of babies who have the top 3 most common pronunciations.*

#### Variations in the Pronunciations of Common Combinations of Chinese Characters

**FIGURE 7:**
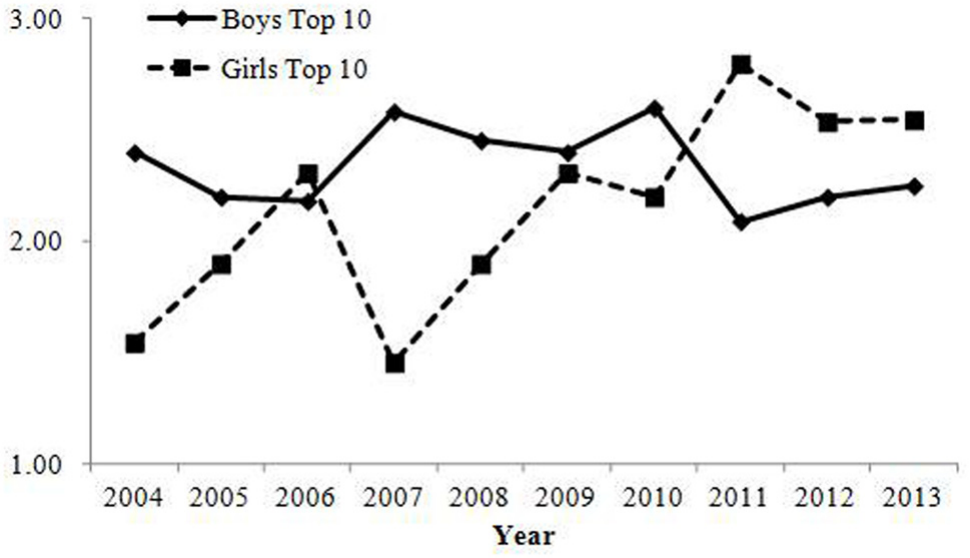
**Average variations of pronunciations in names in the top 10 most common combinations of Chinese characters in Study 2.** Pronunciation variation of girls’ names increased over time. In contrast, variation in boys decreased. Around 2.5 variations seem to be the ceiling.

**FIGURE 8:**
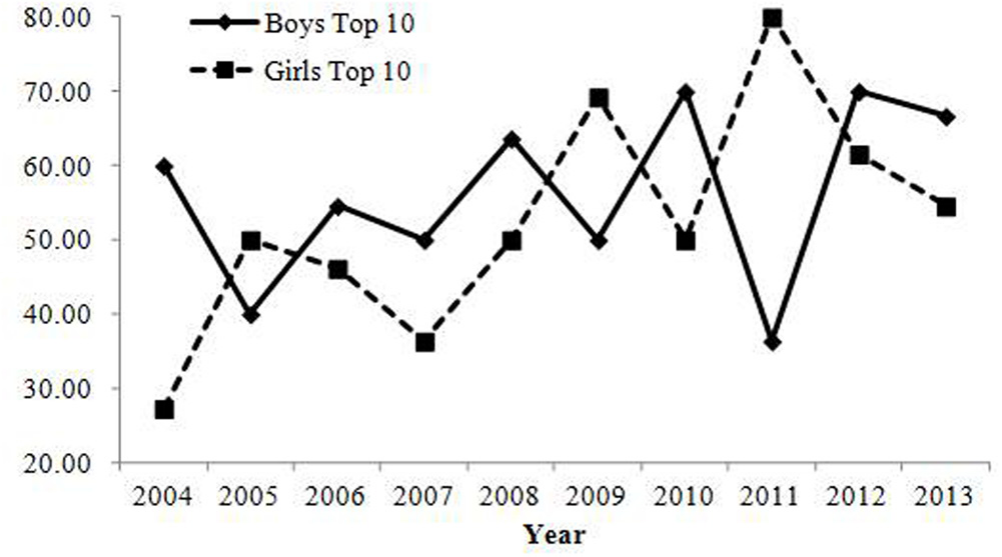
**Percentage of names having more than two pronunciations among the top 10 most common combinations of Chinese characters in Study 2.** The number of names with several pronunciations increased both for boys and girls over the years.

Changes in pronunciation variation are shown in **Table 6**; **Figure 7**. For girls, the correlation between the year and the average variation of readings was positive, which suggests that recently parents in Japan sought uniqueness with regard to the pronunciation of names. Moreover, the correlation between the year and prevalence of names that have more than two readings was positive and had a large annual change (**Table 6**; **Figure 8**). For boys’ names, the correlation between the year and the average variation of readings was negative. Yet, the correlation between the year and the prevalence of names that have more than two readings was positive and its annual change was sufficiently large. The negative correlation between the year and the average variation of readings might be caused by a ceiling effect, which is implied by **Figure 7**. In summary, variations in the pronunciation of the top 10 most common combinations of Chinese characters increased.

**Table 6:** Variations of pronunciation in the top 10 most common combinations of Chinese characters (Study 2).

		2004	2005	2006	2007	2008	2009	2010	2011	2012	2013	Correlation with year (*r*)	Annual change (%)
Boys	Number of combinations^1^	10	10	11	12	11	10	10	11	10	12		
	Total variation^2^	24	22	24	31	27	24	26	23	22	27		
	Average variation^3^	2.4	2.2	2.18	2.58	2.45	2.4	2.6	2.09	2.2	2.25	**-0.18**	—
	Number of combinations having more than two readings^4^	6	4	6	6	7	5	7	4	7	8		
	Rate of combinations having more than two readings (%)^5^	60	40	54.55	50	63.64	50	70	36.36	70	66.67	**0.36**	**1.37**
Girls	Number of combinations^1^	11	10	13	11	10	13	10	10	13	11		
	Total variation^2^	17	19	30	16	19	30	22	28	33	28		
	Average variation^3^	1.55	1.90	2.31	1.45	1.90	2.31	2.20	2.80	2.54	2.55	**0.77**	—
	Number of combinations having more than two readings^4^	3	5	6	4	5	9	5	8	8	6		
	Rate of combinations having more than two readings (%)^5^	27.27	50.00	46.15	36.36	50.00	69.23	50.00	80.00	61.54	54.55	**0.66**	**3.21**

*Correlations and annual changes were calculated by weighting sample sizes. Numbers in bold letters in the columns for “correlation with year” and “annual change” represent scores that are beyond the criterion (more than |0.10| for correlation or more than 0.1% for annual change). ^1^Absolute number of combinations listed among the top 10 common combinations of Chinese characters. ^2^Total number of reading variations included in the top 10 common combinations of Chinese characters. ^3^Total variation divided by number of combinations. ^4^Absolute number of combinations which have more than two readings among the top 10. ^5^Number of combinations having more than two readings divided by number of combinations.*

### Discussion

The results were relatively unstable because sample sizes were small (approximately 4,000 for each gender) compared to those in Study 1 (approximately 20,000 for each gender). If we examined the average scores (e.g., average score of the self-esteem scale), about 4,000 samples is relatively large. Yet, in the current research, we investigated the ranking of common names and their rates. Thus, 4,000 names would yield duplicated names of the same ranks. This would lead to the inconsistent results for female names (the decrease in the rate of common pronunciations over time and the positive relationship between the rate of common pronunciations and the JCS would both be rendered absent). However, in line with Study 1, Japanese parents were more likely to give their boys names with uncommon pronunciations. Further, by investigating changes in the variations of common combinations of Chinese characters and pronunciations, we obtained the result that variations in the pronunciation have increased over time while variations in the written form have decreased. This indicates that parents express uniqueness in pronunciation rather than in writing.

## General Discussion

We examined whether Japanese culture has become more individualistic by investigating temporal changes of baby names as a cultural product. Across two independent samples from an educational service company and an insurance service company, the results were consistent. Specifically, we found that the rates of parents who gave common pronunciations have decreased. In addition, these rates were positively associated with a previously validated index of collectivism (JCS; Yamawaki, 2012) indicating that this index is valid to measure collectivism. In contrast, the rate of parents who gave common Chinese characters (not combination) has increased in Study 1. However, these rates were not positively associated with the JCS, showing that this index is not valid to measure collectivism. Taken together, it is revealed that Japanese culture has put more emphasis on uniqueness and has become more individualistic.

Japanese parents seem to provide unique names by giving common Chinese characters with uncommon pronunciations, which is consistent with an observation from linguistics (Sato, 2007). If parents give names written with common Chinese characters and pronounced in a traditional way, then the rates of common pronunciation should increase. However, they did not increase, but rather decreased. Hence, parents give names with common Chinese characters to their babies but pronounce them uniquely. We obtained results consistent with this conclusion with different measurements. In Study 2, we examined both the selection of Chinese characters and their pronunciation at the same time. We found that pronunciation variations of common combinations of Chinese characters increased over time, suggesting that Japanese parents assign unique pronunciations to the Chinese characters. In order to make names unique, giving both unique Chinese characters and unique pronunciations is not always necessary. People do not express uniqueness in all domains, but do so in a domain where people expect others would notice and recognize the uniqueness. For example, people often display uniqueness by choosing to wear uncommon clothes or have an uncommon hairstyle, but do not express uniqueness in uncommon choices of what dish to eat first at dinner (because people usually do not care what others eat first in everyday situations). Previous studies showed that people with a high need for uniqueness tend to choose a unique favorite professional baseball team, novel, food, color, town, and so on (Okamoto, 1985; Yamaoka, 1993). Yet, they also note that need for uniqueness was not necessarily associated with uniqueness of all targets or domains. They did not find relationships in terms of favorite country, favorite holiday activity, and favorite party or meeting. Giving both uncommon Chinese characters and an uncommon pronunciation simultaneously may make names so unique as to make even an experienced Japanese speaker unable to read them in an appropriate way, which would be undesirable and problematic.

One example of giving common Chinese characters with uncommon pronunciations is the recent phenomenon of some parents in Japan giving English pronunciations to Chinese characters, which was a previously uncommon practice (Sato, 2007; Tamago Kurabu, 2013). For example, Japanese normally pronounce “海 (meaning sea or marine)” as “Kai” or “Umi” but some parents pronounce it as “Marin” in Japanese, after the English word “marine.” Another example that illustrates the practice of pairing common Chinese characters with uncommon pronunciations is that of parents shortening the pronunciation (Sato, 2007; Benesse Corporation, 2012, 2013). For instance, “心 (meaning mind or psyche)” is usually read as “Kokoro” but can be uniquely read as “Ko” or “Koko” and used as such in “心春 (*Ko*haru)” and “心愛 (*Koko*a).” In these ways, Japanese parents seem to provide unique names.

There are at least two reasons why parents in Japan show uniqueness through pronunciation rather than through Chinese characters. First, the number of Chinese characters that Japanese parents can choose from is limited by the Japanese government (2997 characters; Ministry of Justice, 2013). However, they are free (in that there are no legal restrictions against it) to give a novel pronunciation to a given Chinese character, thus creating a unique pronunciation, but not a unique written form (Sato, 2007). Second, uniqueness in the pronunciation might be more effective and prominent than uniqueness in the Chinese character (Sato, 2007; Kobayashi, 2009). That is because uniqueness in the Chinese character is not apparent in daily communication, but uniqueness in pronunciation does manifest in daily interactions. A person’s name may be called out or spoken more frequently than his or her name is written in face-to-face communication. All things considered, recent parents in Japan are more likely to provide unique names by giving uncommon pronunciations with common Chinese characters, which suggests a rise in uniqueness and individualism in Japan^16^^,^^17^. We do not mean that Japanese culture has become entirely individualistic. Rather, we suggest that Japanese culture has gradually become more individualistic.

### Theoretical Implications

To our knowledge, this is the first study to empirically investigate cultural changes in individualism in a Japanese cultural context through the examination of a cultural product. Previous studies have repeatedly shown that European American cultures have become more individualistic over time (e.g., Gentile et al., 2010; Twenge et al., 2010, 2012, 2013; DeWall et al., 2011; Greenfield, 2013; Oishi et al., 2013). However, little research has investigated whether East Asian cultures have become more individualistic by examining cultural products. We used baby names as a cultural product and revealed that not only in European–American cultural contexts, but also in an East Asian cultural context, culture is changing toward greater individualism. Our research empirically demonstrated temporal changes in individualism in an East Asian cultural context, which enables researchers to compare how culture changes across cultures. Therefore, our research contributes to revealing the dynamic aspects of culture. Moreover, cultural change toward greater individualism in historically individualistic cultures and collectivistic cultures may have different implications (Ogihara and Uchida, 2014; Ogihara et al., 2014). Hence, it is important to present empirical data showing the rise of individualism not only in European American cultures, but also in East Asian cultures that have been historically collectivistic (Hofstede, 1980; Triandis, 1995).

We presented empirical data showing the possibility that there has been an increase in unique names in Japan^18^. In present-day Japan, very unique names are sometimes called “*kirakira names* (shining names)” and attract a lot of attention, not only from scientists, but also from lay people. On the one hand, *kirakira names* have some merits, such as having a strong impression and being remembered easily by others. On the other hand, *kirakira names* are so unique and uncommon that others cannot read them properly, which may cause serious problems, such as not being able to be read or called out in the event of an emergency, and even not being hired by a company because of their name. It is said that despite having such costs, a growing number of parents give such *kirakira names* to their children. The practice or custom of naming in Japan – especially the recent practice of providing unique names – has been extensively reported and studied in fields such as sociology and anthropology (e.g., Kobayashi, 2009), linguistics (e.g., Sato, 2007), and Japanese studies (e.g., Sakata, 2006; Ohto, 2012). Yet, most research has not examined empirical data. In this sense, our study connects several disciplines to each other from a perspective of cultural psychology, and contributes to a better understanding of Japanese naming practices and culture by providing empirical evidence.

This study implies that the way to express uniqueness depends on the socio-cultural environment. We suggest that recently, parents in Japan express uniqueness by giving novel pronunciations to common Chinese characters. In China, which uses a related Chinese character-based writing system, parents can choose Chinese characters without restriction but cannot freely assign new pronunciation to them. Therefore, it is said that Chinese parents try to give unique names by providing uncommon Chinese characters (Lafraniere, 2009), which is a different way of expressing uniqueness than in Japan. This example of Chinese naming practices has not yet been examined through empirical evidence, so this would be a future direction and conceptual extension of the current research.

### Practical Implication

We presented empirical data on how parents try to give unique names in Japan. Within the two databases we analyzed, parents are more likely to give unique names by pairing uncommon pronunciations with common Chinese characters. In other words, parents do not seem to give unique Chinese characters to their babies in Japan. Some newspapers, magazines, and people have anecdotally reported parents giving uncommon Chinese characters. However, the present research provided no evidence for this as a mainstream trend, at least in the present samples. Sharing the domain of expressing uniqueness is necessary to efficiently show their uniqueness in the social contexts. In this sense, we provided the important information that uniqueness is expressed more in pronunciation rather than in Chinese characters recently in Japan to persons who try to give unique names.

### Limitations and Future Research

This research examined cultural change over a relatively short period of time (2004–2013). This was because there are many difficulties in collecting older data in Japan unlike in the U.S. where the government (the Social Security Administration) has systematically collected and released the ranking of common baby names of almost an entire population (about 4 million) from 1880 to 2013 (Twenge et al., 2010; Social Security Administration, 2013). However, even in a brief period, we found a meaningful change toward greater individualism. In the future, it would be desirable to examine a longer span of data on Japanese baby names.

Another limitation is the possibility that the result was driven by changes in the sample used, rather than by changes in Japanese culture as a whole. It might be possible that the sample of parents who buy Benesse Corporation’s products and Meiji Yasuda Life Insurance’s products might have changed over time. However, the results were consistent across both studies, which were drawn from independent samples, suggesting that our findings were unlikely to be biased by sample-specific characteristics.

Future studies should also examine the social and psychological consequences of having a unique name. On the one hand, unique names that are not challenging to read might be positively regarded in that such names convey independence and uniqueness. On the other hand, overly unique names such as *kirakira names* may have negative connotations that parents who give *kirakira names* are not well-educated, because of an assumption that names should be readable while *kirakira names* are difficult or impossible to read. Standing out in a not-yet-fully individualistic culture might be related to some negative impacts (Ogihara and Uchida, 2014; Ogihara et al., 2014). To reveal how cultures change and how people adapt to such changes, it is also important to investigate the adaptiveness of unique names.

## Author Contributions

YO conceived and designed this research. YO analyzed the data. All authors interpreted the results. YO wrote the first manuscript and YU provided critical revisions. All authors approved the final version of the manuscript.

## Conflict of Interest Statement

The authors declare that the research was conducted in the absence of any commercial or financial relationships that could be construed as a potential conflict of interest.

## Footnotes

1 It should be noted that some indices did not show an increase of individualism in Japan (Hamamura, 2012). All indices that did not show an increase in individualistic tendency were self-report items of beliefs and values. Previous research has suggested that self-report items can be problematic. For example, people tend to compare themselves with individuals in their culture rather than with those in another culture (the reference-group effect; Heine et al., 2002). Hamamura (2012) already cleared this issue at the area-level and country-level, but has not examined it at the temporal-level. Even if a person has become more individualistic, they might not realize their increase of individualism because they compare their values with contemporary others, rather than with others in the past. In contrast, behavioral measures (i.e., average family size, divorce rate, and proportion of people living in urban areas), which are less likely to suffer from reference group effects, showed an increase of individualism.

2 In terms of the link between naming practices and individualism, seeking uniqueness is thought to be an important component of an individualistic tendency (e.g., Oyserman et al., 2002). Previous research indicated that people in European–American cultural contexts are more likely to choose unique objects, such as an abstract figure or a pen, than people in East Asian cultural context (Kim and Markus, 1999). An additional study done by Kim and Sherman (2008) revealed that a preference for unique objects was positively related to other individualistic tendencies, such as independent self-construal (Singelis, 1994) and need for uniqueness (Snyder and Fromkin, 1977). Although there has been no research examining the relationship between unique names and individualistic tendency in Japan, giving unique names itself should be regarded as an indicator of individualistic behavior across cultures. The extent to which people value uniqueness is different across cultures (Kim and Markus, 1999), but choosing unique objects itself reflects a degree of individualism. Indeed, in Japan, people with a high need for uniqueness tend to choose unique favorites, such as professional baseball teams, novels, foods, colors, and towns (Okamoto, 1985; Yamaoka, 1993).

3 One might assume that the decrease in the rates of common names is because of the increase of absolute variations of names, not the increase of individualistic tendency. But this reasoning is insufficient. If the natural variation explanation is correct, the rate of parents who give common names should continue to decrease over time. However, previous research reported that the rate of common names increased from 1920 to 1949 (Twenge et al., 2010). In other words, while parents were able to choose names from an increasing pool of names, they gave more common names at that time. This period corresponds to World War I and II, so it might be because people became less individualistic during the time. Furthermore, the increase of absolute variations of names can also be an indicator of uniqueness. Yet, the data of absolute variation were not available from the databases used in either the previous or the present research. It would be desirable to see the relationship between the absolute variation of names and uniqueness empirically in the future.

4 In 2014, we finished analyzing data from between 2005 and 2013. In 2015, after finishing this data analysis, Benesse Corporation released a new ranking for 2014. We would have liked to update the analysis by including this new data, but chose not to due to concerns about the validity of the data. On July, 2014, Benesse Corporation accidentally leaked approximately 40,000,000 pieces of personal information, such as names, addresses, and phone numbers. As one of the largest leakages of personal information in Japan, it had a tremendous impact on Japanese society and is being investigated by not only the police, but the Japanese government as well. Due to this incident, the number of Benesse club members was reduced by one-forth (Benesse Holdings, 2015). In addition, people became reluctant to provide personal information to the company. As a result, the sample size in 2014 was two-thirds the size of the average size of samples from between 2005 and 2013. More importantly, it is likely that this incident distorted the quality of the sample, causing a major problem to examine cultural change over time. It is also expected that this incident made Japanese people pay more attention and more reluctant to offer personal information to private companies, which might have distorted the 2014 Meiji Yasuda sample. Therefore, we chose not to analyze the Meiji Yasuda data in 2014 in this paper.

5 Strictly speaking, this ranking for girls’ names included a few *hiragana* names (i.e., written in the *hiragana* syllabary rather than in Chinese characters) among top 50, such as “Sakura” and “Hinata” (the rankings of boys’ names did not include *hiragana* names during this period). *Hiragana* is an additional writing system in the Japanese language, in which letters do not inherently possess any meaning (e.g., large and wing) and have one way to be pronounced. *Hiragana* is similar to Roman alphabet letters, in which the *hiragana* is reliably associated with a pronunciation. For example, “か” is always pronounced “ka.” The numbers of *hiragana* names among top 50 did not change over time (correlation between the number of *hiragana* names and year was -0.01) and the rates did not change over the years (correlation between the rates of *hiragana* names and the year was -0.44, but its annual change was -0.03, which was below the conventional criterion). Hence, the results without *hiragana* names remained the same. Similarly, in Study 2, the ranking of popular combinations of Chinese characters included a few *hiragana* names for girls (for boys, only 1 *hiragana* name was included in 2011; i.e., “Ren”). The rates of *hiragana* names among top 50 did not change (correlation between the rates of *hiragana* names and year was -0.57, but its annual change was -0.05, which was below the conventional criterion), leading to the same result if we excluded *hiragana* names. Therefore, we did not discuss about *hiragana* name further.

6 Unfortunately, the rankings that consider both Chinese characters and pronunciation at the same time were not displayed in this dataset. So it is impossible to distinguish names consisting of the same Chinese characters with different pronunciations (e.g., “大翔” can be read such as “Hiroto,” “Haruto,” “Yamato”) and the same pronunciation with different Chinese characters (e.g., “Koharu” can be written as “心春,” “心晴,” “小春”).

7 In line with previous research (Hamamura, 2012), we did not conduct tests of significance. Sample sizes for each year (i.e., the number of names collected; *N* = approximately 40,000) were sufficiently large. Yet, when we examined change over time, the sample size, namely the number of years investigated, was not sufficiently large (*N* = 9 or 10). Therefore, we used two conventional criteria, following the previous research (Hamamura, 2012).

8 In the previous research by Hamamura (2012), a similar but slightly different calculation was conducted. In it, the difference between the two earliest and the two latest values was divided by the number of years. His calculation is more likely to be biased by plausible errors of the two earliest and the two latest years, whereas the method of calculation used in the current paper more accurately reflects trends over the whole period. Yet, if we applied this previous method to the current data set, the results were similar.

9 It should be noted that the samples of JCS and the name data might be different. It is better to have samples that overlap even when the relationship is examined at the group level.

10 Results of most common names are not appropriate to examine overall cultural changes. If two or three most common names occur at the same rate, the correlations and annual changes are strongly affected. Hence, the top 10 is more adequate to see a broader picture of cultural change. When we see top 20 and 50, their results were almost consistent (see Supplementary Table 1). To avoid redundancy, we report the results of top 10 here.

11 In terms of change in each Chinese character, the pattern looks slightly more prominent for girls. Previous research showed that Hispanic parents exposed to American culture are more likely to give English names (as opposed to Spanish names) to girls than to boys (Sue and Telles, 2007). This finding suggested that parents may hope for their daughters to assimilate to a new socio-cultural context at a faster rate than for their sons. Their research examined the process in which Hispanics assimilate into American culture, but our study investigates the process in which Japanese adapt to changing Japanese culture. The former looks at the process of acculturation to other cultures due to residential relocation or immigration, whereas latter process does not need any transfer. Nevertheless, this is consistent in that one has to adjust oneself or adapt to novel socio-cultural environments. In line with this finding, our result may indicate that during the 2000s in Japan, parents hoped for girls to be unique and independent more than for boys. Yet, if this explanation is correct, there should be gender differences in change of pronunciation, too (but this was not found). We do not have enough data here to discuss gender differences adequately.

12 In this paper, we used individualism and collectivism as antipodes of a single spectrum. This is because most research examining individualism/collectivism at the group-level, rather than at the individual-level, regards individualism/collectivism as a single spectrum (e.g., Hofstede, 1980; Vandello and Cohen, 1999; Hamamura, 2012; Yamawaki, 2012). In fact, a recent paper showed that the dimensionality of individualism/collectivism depends on the level of analysis. Specifically, it was demonstrated that the results of group-level analyses tended to fit a uni-dimensional solution, whereas individual-level tests tended to fit a bi-dimensional view of individualism/collectivism (Taras et al., 2014).

13 Even if we accept the negative relation between the rate of the top 10 most common combinations of Chinese characters for boys’ names and the JCS, the rate itself did not change over time.

14 Even if we accept the negative relationship between the rate of the top 10 most common individual Chinese characters for girls and the JCS, combining this negative relationship with the fact that the rates of common individual Chinese characters have increased over time, it was also shown that Japanese culture has become more individualistic.

15 We checked the similarities and differences in the ranking of Study 1 and 2 to explore how each sample was different. First, the values of average rates of the top 10 most common names in Study 1 and 2 were almost the same (see **Table 2**). In addition to the rates of names, approximately 80% of the top 10 common pronunciations in the Benesse sample were included in top 10 of the Meiji Yasuda sample (Rate of consistence: *M*_boys_ = 0.80, *M*_girls_ = 0.81). And their rank order correlations were relatively high (Boys: average Spearman’s ρ = 0.80, Girls: average Spearman’s ρ = 0.48). Likewise, a consistent rate of common combinations of Chinese characters in the Benesse sample and the Meiji Yasuda sample was high (*M*_boys_ = 0.61, *M*_girls_ = 0.66). And their rank order correlations were relatively high (Boys: average Spearman’s ρ = 0.54, Girls: average Spearman’s ρ = 0.54). These results suggest that, in accordance with our assumption, the samples in Study 1 and 2 are consistent with but independent of each other.

16 One might question whether giving a unique name in the same way as other people do is indeed unique. We think the answer is yes, because the domain of expressing uniqueness should be somewhat shared, as we mentioned earlier. People recognize the domain in which they can express uniqueness, and then they try to show their uniqueness (i.e., providing uncommon names) within that domain. For example, people usually recognize the uniqueness of people who try to show uniqueness by wearing uncommon clothes or an uncommon hairstyle because people share that wearing uncommon clothes or hairstyle is a way of expressing uniqueness. People usually do not think that someone wearing unique clothes is not unique.

17 We think it is difficult to insist that Japanese “people” have become more individualistic. We showed that recently Japanese parents tend to give unique names to their babies, but we do not know why this happens. One plausible explanation is that Japanese individuals may become more individualistic, leading them to seek more uniqueness and provide unique names to their babies. Another possible explanation is that norms or shared beliefs about uniqueness lead Japanese people to give more unique names. Therefore, we write Japanese “culture” has become more individualistic because culture includes people, norm and shared belief. To reveal which layer or level of culture is changing (or not changing) is also an important future issue.

18 It is difficult to strongly insist from this research that the rates of unique name have increased in Japan. Because we were unable to access the raw data of all sampled names (the data we obtained were the rankings of common names and their proportions), in the present research we focused on the rates of common names in order to examine temporal changes in naming practices in Japan. We could not investigate the rates of unique names (e.g., the rates of names which only one child had), the whole distribution, or absolute variations of names. Previous research which used the rates of common baby names had this limitation as well (e.g., Twenge et al., 2010; Varnum and Kitayama, 2011; Grossmann and Varnum, 2015). We found that the rates of common names have decreased over the years. Thus, it can be inferred that the rates of uncommon names have increased. However, we could not investigate directly whether the rates of unique names have increased, so it is possible that they could have an independent trend. We showed that the rates of common names have decreased, which indicates that collectivism has decreased and individualism has increased. Hence, we suggest that the trend of seeking uniqueness has increased as a whole.
