# Supplementary material for: Are common names becoming less common? The rise in uniqueness and individualism in Japan
Source: Front Psychol. 2015 Oct 21;6:1490. doi: 10.3389/fpsyg.2015.01490 (PMC4613833; doi:10.3389/fpsyg.2015.01490)
Supplement: Supplementary file 1 [file Table_1.DOCX]

***Supplementary Material***

**Are common names becoming less common?**

**The rise in uniqueness and individualism in Japan**

Yuji Ogihara*****, Hiroyo Fujita, Hitoshi Tominaga, Syo Ishigaki,

Takuya Kashimoto, Ayano Takahashi, Kyoko Toyohara, & Yukiko Uchida

*****Correspondence: Yuji Ogihara: [ogihara.yuuji.56u@st.kyoto-u.ac.jp](mailto:ogihara.yuuji.56u@st.kyoto-u.ac.jp)

**Supplementary Table 1.** Correlations with year (*r*) and annual changes (%) among each index in Study 1 and Study 2.

|  |  | Study 1 | | Study 2 | |
| --- | --- | --- | --- | --- | --- |
|  |  | Correlation with year (*r*) | Annual change (%) | Correlation with year (*r*) | Annual  change (%) |
| Combination  of Chinese characters | Boys Top 10 | .31 | 0.03 | .22 | 0.04 |
|  | Girls Top 10 | .21 | 0.04 | .02 | 0.00 |
|  | Boys Top 20 | .33 | 0.04 | －.43 | －0**.**05 |
|  | Girls Top 20 | .26 | 0.03 | －.03 | －0.01 |
|  | Boys Top 50 | .52 | 0.09 | .04 | 0.01 |
|  | Girls Top 50 | **.59** | **0.11** | **.46** | **0.11** |
| Individual  Chinese character | Boys Top 10 | **.32** | **0.10** | **－** | **－** |
|  | Girls Top 10 | **.80** | **0.39** | **－** | **－** |
|  | Boys Top 20 | **.46** | **0.15** | **－** | **－** |
|  | Girls Top 20 | **.96** | **0.77** | **－** | **－** |
| Pronunciation | Boys Top 10 | **－.83** | **－0.18** | **－.59** | **－0.11** |
|  | Girls Top 10 | **－.88** | **－0.16** | .29 | 0.09 |
|  | Boys Top 20 | **－.63** | **－0.18** | **－.38** | **－0.11** |
|  | Girls Top 20 | **－.84** | **－0.18** | －.09 | －0.03 |
|  | Boys Top 50 | **－** | **－** | **－.49** | **－0.14** |
|  | Girls Top 50 | **－** | **－** | **－.22** | **－0.10** |

*Note.* Hyphens mean there were no data available from the database. Both scores were weighted by sample size. Numbers in bold letters represent scores that are beyond the criteria both in correlation with year (beyond |.10|) and annual change (beyond 0.1%), which is indicative of change over time.
